# Novel Isoforms of Heat Shock Transcription Factor 1, HSF1γα and HSF1γβ, Regulate Chaperone Protein Gene Transcription*

**DOI:** 10.1074/jbc.M114.570739

**Published:** 2014-05-22

**Authors:** Andreas Neueder, Francesca Achilli, Saliha Moussaoui, Gillian P. Bates

**Affiliations:** From the ‡ Department of Medical and Molecular Genetics, King's College London, London SE1 9RT, United Kingdom and; § Neuroscience Discovery, Novartis Institute for Biomedical Research, CH-4002 Basel, Switzerland

**Keywords:** Alternative Splicing, Heat Shock Protein (HSP), Molecular Chaperone, Stress Response, Transcription Regulation, HSF1, Heat shock Transcription Factor, Heat Shock Response

## Abstract

The heat shock response, resulting in the production of heat shock proteins or molecular chaperones, is triggered by elevated temperature and a variety of other stressors. Its master regulator is heat shock transcription factor 1 (HSF1). Heat shock factors generally exist in multiple isoforms. The two known isoforms of HSF1 differ in the inclusion (HSF1α) or exclusion (HSF1β) of exon 11. Although there are some data concerning the differential expression patterns and transcriptional activities of HSF2 isoforms during development, little is known about the distinct properties of the HSF1 isoforms. Here we present evidence for two novel HSF1 isoforms termed HSF1γα and HSF1γβ, and we show that the HSF1 isoform ratio differentially regulates heat shock protein gene transcription. *Hsf1*γ isoforms are expressed in various mouse tissues and are translated into protein. Furthermore, after heat shock, HSF1γ isoforms are exported from the nucleus more rapidly or degraded more quickly than HSF1α or HSF1β. We also show that each individual HSF1 isoform is sufficient to induce the heat shock response and that expression of combinations of HSF1 isoforms, in particular HSF1α and HSF1β, results in a synergistic enhancement of the transcriptional response. In addition, HSF1γ isoforms potentially suppress the synergistic effect of HSF1α and HSF1β co-expression. Collectively, our observations suggest that the expression of HSF1 isoforms in a specific ratio provides an additional layer in the regulation of heat shock protein gene transcription.

## Teaser

**Background:** HSF1 is the major eukaryotic transcription factor that regulates expression of HSP genes.

**Results:** We identify two novel HSF1 isoforms and show that HSF1 isoforms differentially regulate chaperone gene transcription.

**Conclusion:** HSF1 isoforms work synergistically, and the ratio of HSF1 isoforms determines chaperone gene transcription levels.

**Significance:** Our findings unravel an additional layer of chaperone gene regulation through modulation of HSP expression by HSF1 isoform ratios.

## Introduction

Organisms often face temperature changes in environmental conditions. Molecular chaperones, also termed heat shock proteins, are induced as a cellular response to this proteotoxic stress. Eukaryotes possess a family of transcription factors that regulate this stress response program: the heat shock transcription factors. To date seven members of this family have been identified: HSF1,^3^ HSF2, HSF3, HSF4, HSF5, HSFY, and HSFX (1). There is very little information about HSF3, HSF5, HSFX, and HSFY as these are mostly yet to be characterized (2). In contrast to HSF1, HSF2 is not activated by a heat shock (3, 4). However, it has been shown to co-localize (5) and interact with HSF1 (5–7) and to mediate transcription of heat shock element-containing genes (6, 8). HSF2 can be induced by proteasome inhibition (9, 10), and it was shown to be required for development and cellular differentiation (4, 11–14). HSF4 is also not activated by heat shock and probably does not act as a classical inducible transcription factor (15), yet it functionally interacts with HSF1 during lens development (16).

HSF1, HSF2, and HSF4 are all known to be expressed as two isoforms with tissue specific patterns of expression (17, 18). Unsurprisingly, this complex expression profile of heat shock factor isoforms has been shown to influence the level of transcription of chaperone genes. The two HSF4 isoforms (HSF4a and HSF4b) have opposing effects on the basal levels of chaperone genes (17). HSF4a attenuates the constitutive expression of chaperones, as well as induction after heat shock, probably by competing with HSF1 for binding at heat shock elements (17, 19). On the other hand, HSF4b can activate transcription of heat shock response genes and at least partially substitute for loss of HSF1 (17). The two isoforms of HSF2 (HSF2α and HSF2β) act mostly as transcriptional regulators of the HSF1-dependent activation of heat shock protein (HSP) genes (7, 20). Recently it was shown that, in response to proteasome inhibition, the HSF2β isoform, in particular, negatively regulates the HSF1-dependent induction of HSP genes (8).

The two known HSF1 isoforms were shown to be generated from the same gene by alternative splicing (21). Inclusion of exon 11 produces the longer α isoform, whereas exclusion leads to the shorter β isoform. Alternative splicing has also been proposed as the mechanism by which multiple HSF isoforms are generated in other species (22–24). In mammals *HSF1* mRNA does seem to be constitutively expressed and transcription is not induced by heat stress (25). In contrast, fish have two isoforms for the HSF1 homologue, and interestingly, their expression is inducible by various stresses (26–29).

Despite their identification over 20 years ago (2), very little is known about the functional differences of HSF1 isoforms, in particular under heat stress conditions. In this study, we functionally characterize the known HSF1 isoforms and their transcriptional potential in the mouse. We also describe two additional HSF1 isoforms and analyze the time course of their activation by post-translational modifications and nuclear-cytoplasmic transport kinetics. We were able to show that the two novel isoforms are ubiquitously expressed and can be translated into proteins. We also demonstrate that the individual HSF1 isoforms are post-translationally modified to a similar extent, but are exported from the nucleus, or degraded after heat shock, with differential kinetics. Finally, we show that the HSF1 isoform ratio determines the level of heat shock protein gene expression.

## EXPERIMENTAL PROCEDURES

### 

#### 

##### Mouse Maintenance, Breeding, and Genotyping

*Hsf1* knock-out mice (C;129-*Hsf1*^tm1Ijb^/J) (30) were purchased from The Jackson Laboratory (strain number 010543). They were bred to and maintained on a (CBA × C57BL/6) F1 background (B6CBAF1/OlaHsd, Harlan Olac, Bicester, UK). All experimental procedures were approved by the King's College London Ethical Review Committee and performed in accordance with United Kingdom Home Office regulations. All animals had unlimited access to food and water and were subject to a 12-h light/dark cycle. Housing conditions and environmental enrichment were as described previously (31). Genomic DNA was isolated from an ear punch. PCR was performed with primers Hsf1KoF (5′-AGACCTGTCCTGTGTGCCTAGC), Hsf1KoR (5′-CAGGTCAACTGCCTACACAGACC), Neo3 (5′-AGGACATAGCGTTGGCTACCCGT), and Neo4 (5′-GCCTGCTATTGTCTTCCCAATCC) for 35 cycles (95 °C 25 s, 60 °C 20 s, 72 °C 45 s) with the GoTaq system (Promega).

##### HSP990 Treatment

NVP-HSP990 ((*R*)-2-amino-7-((*R*)-4-fluoro-2-(6-methoxypyridin-2-yl)phenyl)-4-methyl-7,8-dihydropyrido[4,3-d]pyrimidin-5(6*H*)-one) (32) was obtained from Novartis Pharma AG (Basel, Switzerland). The drug was formulated in 0.2% methyl cellulose in 0.9% saline solution (water with 0.9% NaCl) as vehicle. The HSP990-vehicle mixture was sonicated in a water bath to create a uniform suspension with very small particle size. The drug was freshly prepared for each round of treatment and administered by oral gavage, with thorough mixing between dosing to ensure a homogenous suspension. Mouse tissue was snap-frozen in liquid nitrogen and stored at −80 °C.

##### Generation of Primary Cell Lines

Heterozygous *Hsf1* transgenic animals were interbred to obtain homozygous *Hsf1* knock-out and wild type littermates. Tissue from two ear punches from the same animal for each cell line was sterilized with 70% ethanol (v/v), chopped into small pieces, and put in a 12.5-ml flask with fibroblast medium (DMEM, high glucose, GlutaMAX with pyruvate, 15% fetal bovine serum, 1× minimum essential medium nonessential amino acids, and penicillin/streptomycin). The tissue pieces were incubated at 37 °C until they attached and cells started to migrate. This was defined as passage 0. Cells were subsequently incubated with trypsin solution and transferred into fresh medium. All experiments were performed between passages 3 and 6.

##### Heat Shock Treatment

Flasks or multiwell plates were sealed with Parafilm and submerged in a water bath. Heat shock treatment was 42 °C for 45 min unless otherwise stated. Flasks or plates were put back at 37 °C to let the cells recover from heat shock. Non-induced cells were maintained at 37 °C.

##### RT-PCR and Quantification of Hsf1 Isoform Expression

RNA from tissue or cells was extracted using QIAZOL together with RNeasy mini kits (Qiagen) according to the manufacturer's instructions. 1 μg of RNA was reversed-transcribed using an oligo(dT) (T_18_) oligonucleotide. 5% of the cDNA reaction was used as template for the isoform RT-PCR assay with primers HSF1iso-t2_f (5′-TCAGCGTAGCCTGCCTAGACAA), HSF1iso-t2_r (5′-GCTCTTGTGGAGACAGAAGCTCC), GAPDH_new_r (5′-GTGGGTGCAGCGAACTTTATT), and GAPDH_111_f (5′-CACTGAGCATCTCCCTCACAATT). The PCR conditions were: 95 °C for 2 min, 30 cycles of 95 °C 20 s, 60 °C 20 s, 72 °C 45 s, and a final 6 min at 72 °C. PCR products were separated on a 3% MetaPhor agarose gel (Lonza). Bands were digitally visualized on a Benchtop UV transilluminator (UVP) and quantified using the Image Studio Lite version 3.1 software (LI-COR).

##### Generation of Hsf1 Isoform Expression Plasmids, Luciferase Assay Plasmids, and Transfections

*Hsf1* isoforms were amplified from oligo(dT) reverse-transcribed wild type RNA. Primers for untagged isoforms were mHSF1_kozak_f (5′-GCCGCCATGGATCTGGCCGTGGGCC), and primers for FLAG-tagged isoforms were mHSF1_FLAG_kozak_f (5′-GCCGCCATGGATTACAAGGATGACGATGACAAGGGTCTTTTAATGGATCTGGCCGTGGGCC), both together with mHSF1_rev (5′-CTAGGAGACAGTGGGGTCCTTGG). PCR products were cloned into pCR8/GW-TOPO vector (Life Technologies). Both untagged and FLAG-tagged versions of *Hsf1*α and *Hsf1*γα were obtained. To generate the other isoforms, the α/β-cassette (exon 10 to exon 13) was exchanged by PstI/SacI digest. All plasmids were verified by sequencing, with National Center for Biotechnology Information (NCBI) accession number NC_000081.6 from base pairs 76477389 to 76500978 being used as a reference. *Hsf1*α isoforms contain a silent mutation in exon 9 (A1068G from the ATG start codon), *Hsf1*γα isoforms contain a silent mutation in exon 11 (G1323A from the ATG start codon), and *Hsf1*β and *Hsf1*γβ have no mutations. The gateway entry vectors were subsequently inserted into pT-RExT-DEST30 to give the final expression plasmids. All plasmids are driven by the CMV promoter. The firefly luciferase gene was cloned with primers FFLuc_for (5′-GCGCTCGAGAAGCTTGGATCCGCGGTACCATGGAAGACGCCAAAAACATAAAG) and FFLuc_rev (5′-CGCGAGCTCTTACAATTTGGACTTTCCGCC) into pcDNA3 to give plasmid pcFFLuc. The *Hspa1a/b* promoter was cloned into pcFFLuc with primers hsp70_for (5′-GCGCTCGAGCCCCAGAAACCTCTGGAGAGT) and hsp70_rev (5′-CGCGGTACCGCGCTCTGCTTCTG). All plasmids were transfected with jetPRIME (Polyplus-transfection SA) according to the manufacturer's instructions. The amount of DNA for each transfection, including the isoform combinations, was kept constant (2 μg per 6-well plate, 1 μg per 12-well plate). Until otherwise noted, all experiments were performed 48 h after transfection.

##### Antibodies and Western Blotting

Anti-HSF1γ antibody was raised against peptide LARAPQMSGVARLFPCPSS in rabbit (Davids Biotechnologie GmbH) and used 1:150 in TBS-T (50 mm Tris-Cl, pH 7.4, 150 mm NaCl, 0.1% (w/v) Tween 20) for 5 h at room temperature. HSF1γ preimmune serum was used at 1:5000 in TBS-T for 5 h at room temperature. Other antibodies and dilutions were GAPDH (ab9485, Abcam) 1:1000, HSF1 (ab81279, Abcam) 1:500, HSF2 (sc-13056, Santa Cruz Biotechnology) 1:75, HSF3 (SAB2105376, Sigma) 1:100, HSF4 (sc-19860, Santa Cruz Biotechnology) 1:200, β-actin (ACTB) (sc-47778, Santa Cruz Biotechnology) 1:10000, HSP70 (SPA-810, StressGen) 1:1000, HSP40 (SPA-400, StressGen) 1:1000, and HSP25 (SPA-801, StressGen) 1:1000. Blocking buffer was 5% (w/v) skimmed milk powder in TBS-T. Secondary antibodies were purchased from LI-COR, and Western blots were visualized on an Odyssey Sa (LI-COR) and analyzed with the Image Studio Lite version 3.1 software (LI-COR). Membranes were stripped from bound antibody by incubation at 55 °C for 30 min with three changes of buffer during this time (62.5 mm Tris-Cl, pH 6.6, 100 mm β-mercaptoethanol, 2% SDS). After stripping, membranes were extensively washed in TBS-T and reblocked.

##### Immunoprecipitation

Mouse tissue was homogenized in ice-cold lysis buffer (PBS (Life Technologies), 1% (w/v) Triton X-100, 1 mm dithiothreitol, 5 mm EDTA, Complete protease inhibitors (Roche Applied Science)), used immediately, and never frozen. Sample concentration was determined by measuring absorbance at 280 nm or using a BCA assay (Thermo Scientific). About 5 mg of total protein was incubated with either 3 μg of anti-HSF1 (Abcam) antibody or 3 μg of rabbit IgG (2729S, Cell Signaling Technology). Immunoprecipitation (IP) was carried out overnight at 4 °C. On the next day, 10 μl of preblocked (0.5 mg/ml bovine serum albumin) magnetic Dynabeads protein G beads (Life Technologies) was added, and the IPs were incubated for another 1.5 h at 4 °C. IPs were washed four times with 0.5 ml of cold lysis buffer.

##### Immunofluorescence

Cells were grown directly on poly-l-lysine-coated coverslips in 12-well plates. Cells were washed with PBS and fixed for 16 min at room temperature with 4% formaldehyde (Sigma) in PBS. Cells were washed twice with PBS followed by permeabilization with 0.1% Triton X-100 (w/v) in PBS for 5 min. Cells were washed with PBS and blocked for 20 min at room temperature with 3% skimmed milk powder (w/v) in PBS. Coverslips were incubated overnight at 4 °C with anti-FLAG antibody (2368P, New England Biolabs) 1:220 in block buffer with 0.5% Tween 20. Cells were washed three times with TBS-T and incubated for 1 h at room temperature with A488-coupled goat-anti-rabbit secondary antibody (1:1000 in PBS with 1 μg/ml DAPI). Cells were washed three times with TBS-T and mounted in VECTASHIELD mounting medium (Vector Laboratories). Images were taken on an Eclipse Ti-E inverted CSU-X1 spinning disk confocal with the NIS-Elements C software (Nikon).

##### Quantitative PCR

TaqMan RT quantitative PCR, and the evaluation of the data was performed as described previously (33). The quantitative PCR assays for GAPDH and β-actin were purchased from Primer Design. Primers and probes (5′-FAM, 3′-TAMRA) for *Hspa1a/b*, *Dnajb1*, and *Hspb1* were purchased from Eurofins MWG. Sequences are as follows: Hspa1a/b-F, 5′-GGTGGTGCAGTCCGACATG; Hspa1a/b-R, 5′-TTGGGCTTGTCGCCGT; Hspa1a/b-P, 5′-CACTGGCCCTTCCAGGTGGTGAA; Dnajb1-F, 5′-CCCCATGCCATGTTTGCT; Dnajb1-R, 5′-GCGCTGCCCAAAAAAGG; Dnajb1-P, 5′-TCTTCGGTGGCAGAAACCCCTTTGA; Hspb1-F, 5′-CACTGGCAAGCACGAAGAAAG; Hspb1-R, 5′-GCGTGTATTTCCGGGTGAAG; and Hspb1-P, 5′-ACCGAGAGATGTAGCCATGTTCGTCCTG.

##### Luciferase Assay

Cells were seeded in white 96-well plates (Thermo Scientific) and transfected with 100 ng of each *Hsf1* expression plasmid, HSP promoter-driven firefly luciferase (*Photinus pyralis*), and thymidine kinase promoter-driven *Renilla* (*Renilla reniformis*) luciferase. The amount of DNA for each transfection, including the isoform combinations, was kept constant. The Dual-Luciferase reporter assay system (Promega) was used according to the manufacturer's instructions. Luminescence signals were read on an Orion II microplate luminometer (Berthold Detection Systems).

##### Statistics

Data were screened for outliers using Grubb's test (GraphPad). Statistical significance was calculated using SPSS (IBM). Analysis of variance with Tukey's post hoc test was used to calculate significance. *p* values less than 0.05 were considered as statistically significant.

## RESULTS

### 

#### 

##### Two Novel HSF1 Isoforms Are Expressed in Various Mouse Tissues

**FIGURE 1.**
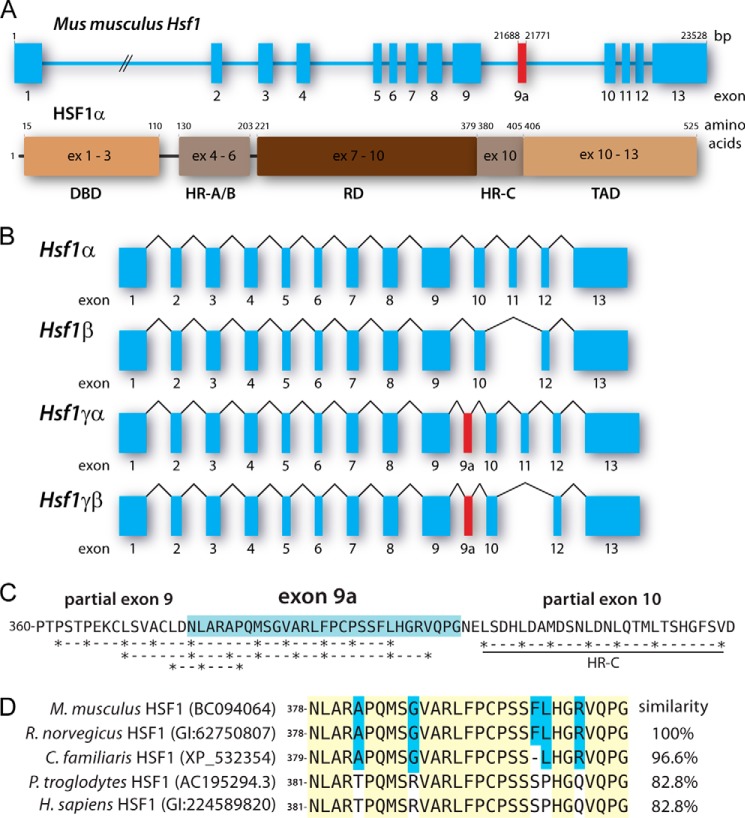
*A*, schematic of murine *Hsf1* gene and HSF1α protein structure (NCBI accession 15499). Exon (*ex*) 9a, highlighted in *red*, encodes the *Hsf1* γ (HSF1γ) isoforms and is a 84-bp exon. The DNA binding domain (*DBD*) is encoded by exons 1–3, heptad repeats A and B (*HR-A/B*) are encoded by exons 4–6, the regulatory domain (*RD*) is encoded by exons 7–9 and the first two amino acids of exon 10, the heptad repeat C (*HR-C*) is encoded by exon 10, and the transcription activation domain (*TAD*) is encoded by the last seven amino acids of exons 10 and exons 11–13. *B*, the mRNA structures of all four possible *Hsf1* isoforms. Exons are shown as *boxes*, and introns are shown as *lines*. Exon 11 defines the α and β isoforms, and exon 9a defines the γ isoforms. *C*, possible hydrophobic heptad repeats encoded by exon 9 and 9a. The additional amino acid sequence of the γ isoforms is highlighted in *blue. D*, the exon 9a encoded amino acid sequence is conserved in higher eukaryotes. The NCBI protein accession number is given in *brackets* after the species name. Amino acids highlighted in *yellow* are fully conserved; *blue background* depicts partial conservation. Murine exon 9a was used as reference, and the similarity of the other sequences was calculated accordingly. Genus abbreviations: *M. musculus*, *Mus musculus*; *R. norvegicus*, *Rattus norvegicus*; *C. familiaris*, *Canis lupus familiaris*; *P. troglodytes*, *Pan troglodytes*; *H. sapiens*, *Homo sapiens*.

We first discovered the additional exon 9a, which gives rise to the two novel HSF1 isoforms, during the cloning of a full-length *Hsf1*α transcript. This novel exon was present in ∼10% of the clones that were analyzed. Sequencing allowed us to map this exon to a genomic sequence of *Hsf1* at base pairs 21688 to 21771 (NCBI accession 15499) from the transcription start site (Fig. 1*A*). We termed the two novel transcripts *Hsf1*γα and *Hsf1*γβ, with *Hsf1*γα containing exon 9a and exon 11 and *Hsf1*γβ containing only exon 9a. The genetic structure of the four *Hsf1* isoforms is shown in Fig. 1*B*. Exon 9a contains 84 bp and consequently does not cause a frameshift when included. The amino acid sequence encoded by exon 9a is shown in Fig. 1*C*. In Fig. 1*C*, the *asterisks* mark hydrophobic amino acids in a potential heptad repeat spacing. Although cDNA clones containing exon 9a sequences can be found in the NCBI database, this sequence was never assigned an official *HSF1* exon status. A BLAST analysis of the exon 9a amino acid sequence against the NCBI database for mammals found that the murine exon 9a is highly similar to other mammalian homologues (Fig. 1*D*).

**FIGURE 2.**
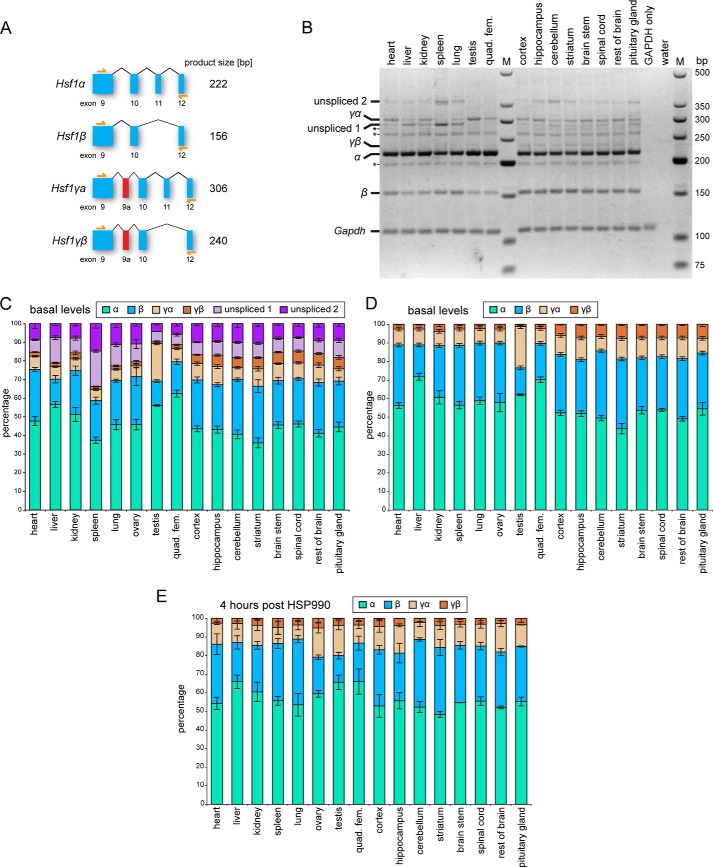
***Hsf1* isoform ratios in mouse tissue.** *A*, schematic of the RT-PCR assay to quantify *Hsf1* isoform ratio. Primers (*yellow arrows*) bind in exons 9 and 12 and give distinct product lengths for each isoform. *B*, representative image of an RT-PCR assay showing *Hsf1* isoform ratios in different mouse tissues. Tissues were taken from 8-week-old (CBA × C57BL/6) F1 wild type mice. All visible bands are specific for *Hsf1* sequences and were confirmed by sequencing. *Asterisks* mark *Hsf1* isoforms that contain some retained introns, but because they show very similar levels in all tissues, were not quantified. *Unspliced 1* (*Hsf1*α with retained intron 11, 292 bp) and *unspliced 2* (*Hsf1*α with retained introns 10 and 11, 369 bp) represent the major immature isoforms and are quantified in *C*, together with *Hsf1*α (α), *Hsf1*β (β), *Hsf1*γα (γα), and *Hsf1*γβ (γβ). GAPDH was used as loading control. Marker (*M*) is low molecular weight marker (New England Biolabs) *quad. fem.* = quadriceps femoris. *C*, quantification of *Hsf1* isoform ratios in different mouse tissues. The intensity of the GAPDH band was used to average the band intensities from different mice. The sum of the normalized band intensities of all *Hsf1* isoforms for each tissue was set to 100%. The data shown are the mean intensity (*n* = 6; *n* = 3 for ovary and testis) ± S.E. *D*, same as in *C*, but the unspliced isoforms were not considered and the sum of the band intensities for the four main isoforms was set to 100%. *E*, same as in *D*, but mice were treated with 12 mg/kg HSP990 4 h before tissues were taken. Data are mean (*n* = 2) ± S.E.

To further analyze the expression of *Hsf1* isoforms in different tissues, we used an RT-PCR assay, which can distinguish between all four isoforms (Fig. 2*A*). This was run on cDNA from six peripheral tissues and eight brain regions from 8-week-old wild type mice (Fig. 2*B*). The identity of the isoform bands was confirmed by sequencing. In addition to the four major *Hsf1* isoforms (α, β, γα, γβ), we could detect multiple additional bands, all specific for *Hsf1* sequences as confirmed by sequencing. The *three asterisks* in Fig. 2*B* mark isoforms, with retained partial intronic sequences that were not differentially expressed across tissues. In contrast, the two immature isoforms labeled as unspliced 1 (*Hsf1*α with retained intron 11) and unspliced 2 (*Hsf1*α with retained introns 10 and 11) did show differences in expression levels between tissues (Fig. 2*B*). The expression levels of the four mature *Hsf1* isoforms and the two immature isoforms were quantified in Fig. 2*C*. The intensities of all bands in each tissue were normalized to the GAPDH band intensity, and the mean expression of the normalized intensities between experiments was calculated. Either the sum of all *Hsf1* isoform band intensities for each tissue was set to 100% (Fig. 2*C*), or only the sum for the four major *Hsf1* isoform was set to 100% (Fig. 2, *D* and *E*). Some of the peripheral tissues showed a very high level of the immature *Hsf1* isoforms (*e.g.* spleen in Fig. 2*C*, *unspliced 1* and *2*). *Hsf1* isoform ratios in brain regions were much more homogenous. Overall, the γ isoforms constituted about 10% of all *Hsf1* isoforms in the peripheral tissues and about 15% in the brain regions. The proportion of the *Hsf1*γα isoform was quite variable in peripheral tissues, with testes showing exceptionally high levels of about 22% (Fig. 2*D*). The Hsf1γβ isoform was expressed at almost 3-fold higher levels in brain regions than in peripheral tissues (Fig. 2*D*, 7.0% brain *versus* 2.4% periphery). The periphery also showed a slightly lower percentage of α isoforms and a slightly higher level of β isoforms. These *Hsf1* isoform ratios remained highly comparable in tissues from mice that had been treated with HSP990 to induce the heat shock response (compare Fig. 2, *D* and *E*). The only significant difference (analysis of variance, *p* < 0.05) was a lower percentage (about 2-fold) of the *Hsf1*γβ isoform in the hippocampus, striatum, and brain stem after HSP990 dosing (compare Fig. 2, *D* and *E*).

##### The Hsf1γ Isoforms Are Translated into Protein in Vivo

**FIGURE 3.**
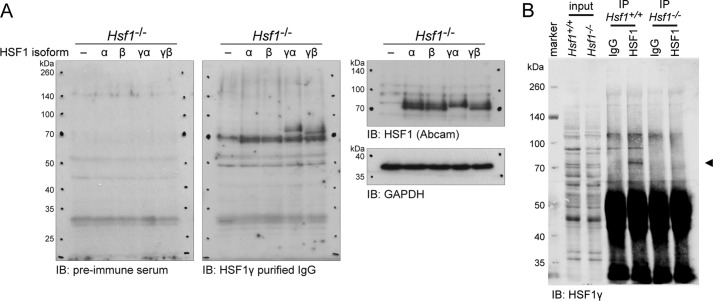
**The *Hsf1*γ isoforms are translated into protein *in vivo*.** *A*, verification of the anti-HSF1γ antibody raised against the exon 9a peptide. An *Hsf1* knock-out cell line (*Hsf1*^−/−^) was transfected with either vector (−) or the four HSF1 isoforms (α, β, γα, γβ). Proteins were extracted and separated by SDS-PAGE. The membrane was first incubated with HSF1γ preimmune serum. After image acquisition, the membrane was stripped and incubated with anti-HSF1γ purified IgG. Thirdly, the blot was stripped again, cut, and incubated with anti-HSF1 (Abcam), or GAPDH, respectively. *IB* = immunoblot detection antibody. *B*, IP of HSF1 from mouse testes. Testes from wild type (*Hsf1*^+/+^) and knock-out (*Hsf1*^−/−^) mice were homogenized, and HSF1 was immunoprecipitated as described under “Experimental Procedures.” Following Western blotting, the membrane was cut at about 60 kDa, but each part was incubated with identical conditions and reassembled for image acquisition. HSF1γ isoforms were detected with anti-HSF1γ purified IgG (◀) in the IP from wild type, but not from knock-out mice.

To determine whether the two novel *Hsf1* isoforms were translated into protein *in vivo*, we generated an antibody against the exon 9a peptide. Validation of the anti-HSF1γ antibody in an *Hsf1* knock-out cell line showed that it indeed recognized the two HSF1γ isoforms, but not HSF1α nor HSF1β (Fig. 3*A*, *middle panel*). A commercially available antibody against HSF1 (ab81279, Abcam), which was raised against full-length HSF1 (epitope: amino acids 288–439), recognized all four isoforms (Fig. 3*A*, *right panel*). We detected the highest level of total *Hsf1*γ expression with our RT-PCR assay in testis. Therefore, we immunoprecipitated HSF1 from the testes of 8-week-old wild type and *Hsf1* knock-out mice and probed with the anti-HSF1γ antibody (Fig. 3*B*). We could detect a signal in the IP of HSF1 in wild type mice, but not in the knock-out mice. The very small difference in size of only 2.3 kDa between HSF1γα and HSF1γβ made it impossible to separate the two isoforms on a gel. This paradigmatic result demonstrates that HSF1γ isoforms are translated into protein *in vivo*.

##### Heat Shock Treatment Induces the Hypershift of HSF1 Isoforms, Indicating Activation through Post-translational Modifications

**FIGURE 4.**
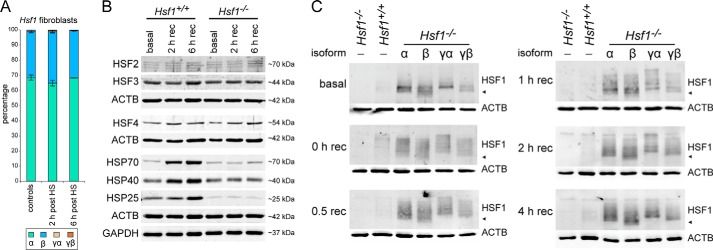
**HSF1 isoforms are post-translationally modified.** *A*, *Hsf1* isoform ratio in wild type primary fibroblast cell lines. The experiment was performed as described in the legend for Fig. 2. Data are mean (*n* = 4) ± S.E. *HS* = heat shock. *B*, characterization of the established primary cell lines. Wild type (*Hsf1*^+/+^) and knock-out (*Hsf1*^−/−^) fibroblast lines were analyzed for protein expression levels of HSF2, HSF3, HSF4, HSP70 (*Hspa1a/b*), HSP40 (*Dnajb1*), and HSP25 (*Hspb1*). GAPDH and β-actin (*ACTB*) were used as loading controls. *rec* = recovery. *C*, time course of HSF1 isoform activation. Wild type and knock-out fibroblast lines were transfected with vector (−) and the knock-out line in addition to the four HSF1 isoforms (α, β, γα, γβ). Cells were kept at 37 °C (basal) or heat-shocked and recovered for the indicated time period (0–4 h rec) at 37 °C before Western blot analysis. HSF1 isoforms were detected with anti-HSF1 (Abcam). β-Actin (*ACTB*) was used as a loading control. ◀ indicates ∼70 kDa.

We next developed a cell model system to characterize the individual HSF1 isoforms in more detail. We established two fibroblast lines each from wild type and from *Hsf1* knock-out mice. Using the same RT-PCR assay as before, we analyzed the isoform ratios in the wild type fibroblast lines (Fig. 4*A*). Only miniscule amounts of the *Hsf1*γ isoforms could be detected. The ratio of *Hsf1*α to *Hsf1*β was about 2 to 1 and did not significantly change after heat shock (Fig. 4*A*). Furthermore, we analyzed the expression of HSF2, HSF3, and HSF4 in the fibroblast lines and found that there was no apparent change in the basal expression of any HSFs between the wild type and *Hsf1* knock-out lines, nor after heat shock (Fig. 4*B*). On the other hand, the induction of heat shock proteins, as reported previously (30), depends on the presence of HSF1 (Fig. 4*B*). Interestingly, HSP70 (*Hspa1a/b*) and HSP40 (*Dnajb1*) levels under basal conditions were not affected by the *Hsf1* knock-out (Fig. 4*B*, *basal lanes*). Because the duplicate cell lines for both the wild type and the *Hsf1* knock-out cell lines were identical regarding isoform ratio and induction of heat shock proteins, we only focused on one line for each genotype in the following experiments.

Individual isoforms expressed in the *Hsf1* knock-out cell line resulted in readily detectable signals at the same apparent molecular weight as in the wild type cell line (Fig. 4*C*, *basal*). This implied a much higher expression level of the individual HSF1 isoforms when compared with their endogenous counterparts, most likely because their expression was under the control of the very strong CMV promoter. However, all isoforms were expressed to a similar level when compared with each other (Fig. 3*A*, *right panel*, and Fig. 4*C*). HSF1 activation by heat stress induces a shift of the HSF1 bands toward higher molecular weight (hypershift) due to extensive post-translational modifications (34). A time course of activation for each of the individual HSF1 isoforms showed that all isoforms, as well as the wild type signal, were hypershifted immediately after heat shock (Fig. 4*C*, *0 h rec lane*). This hypershift was still visible even 4 h after heat shock, although at this time point, the very high molecular weight signals had diminished, indicating a deactivation of HSF1 (Fig. 4*C*). Taken together, all HSF1 isoforms were extensively post-translationally modified after heat shock and were activated with similar kinetics when compared with each other and with the wild type situation.

##### HSF1 Isoforms Are Imported into the Nucleus after Heat Shock Treatment

**FIGURE 5.**
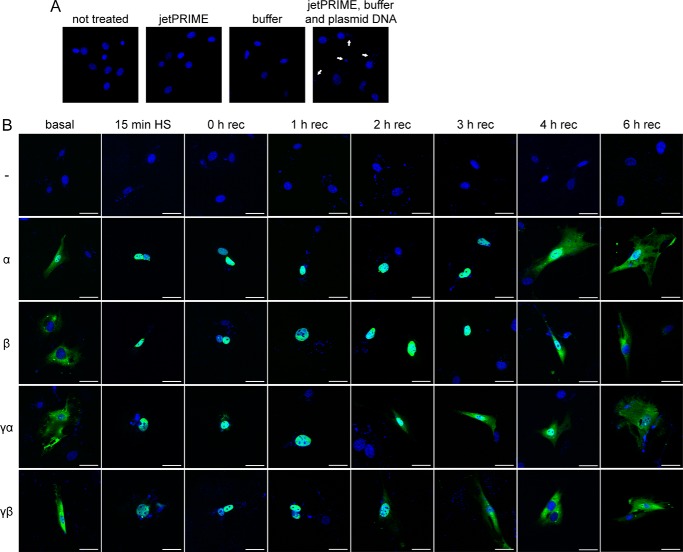
**HSF1 isoforms show different nuclear export or degradation kinetics after heat shock.** *A*, DAPI stain (*blue*) of *Hsf1* knock-out cells with different transfection conditions. Cells were completely untreated (*not treated panel*), transfected with only the jetPRIME reagent (*jetPRIME panel*) or only with the buffer (*buffer panel*), or transfected with both together with plasmid DNA (*jetPRIME, buffer and plasmid DNA panel*). The *white arrows* indicate the appearance of DAPI-positive small puncta when DNA is transfected. *B*, immunofluorescence of HSF1 isoforms after heat shock (*HS*). Cells were either kept at 37 °C (*basal panel*) or heat-shocked for 45 min at 42 °C and recovered for the indicated time period (0–6 h rec) at 37 °C. The second column shows cells that were only heat-shocked for 15 min (*15 min HS panel*) at 42 °C and immediately fixed. Images are shown as merged color with DAPI in *blue* and anti-FLAG (FLAG-tagged HSF1 isoforms) in *green. White bars* correspond to 50 μm. *rec* = recovery.

We used immunofluorescence to study the intracellular localization of HSF1 isoforms in non-induced conditions or after heat shock. Our attempt to detect endogenous HSF1 with different antibodies (Abcam (ab81279, ab52757, ab2923); Santa Cruz Biotechnology (sc-17757, sc-9144, sc-30443); StressGen (ADI-SPA-901); NeoMarkers (Ab-3 10H8)), in a variety of conditions, was unsuccessful as in each case, we observed a positive signal in the *Hsf1* knock-out fibroblasts (data not shown). Hence, we used the N-terminal FLAG epitope-tagged HSF1 isoforms to analyze their intracellular distribution. When we analyzed the transfected fibroblast lines, we observed small DAPI-positive foci appearing (Fig. 5*A*, *right panel*, *white arrows*). However, this did not occur when DNA was omitted, *i.e.* when only the components of the transfection system were used (Fig. 5*A*, *jetPRIME* and *buffer panels*). We concluded that the polyethylenimine-DNA complexes were very stable and could be stained with DAPI. In the non-induced situation, the HSF1α isoform showed a relatively high level of signal in the nucleus, whereas the other isoforms showed almost exclusive cytoplasmic staining (Fig. 5*B*, *basal panel*). Consistent with the observed hypershift due to extensive post-translational modifications (Fig. 4*C*), the HSF1 isoforms could only be detected in the nucleus immediately after heat shock (Fig. 5*B*, *0 h rec panel*), and a shorter heat shock of only 15 min at 42 °C was also sufficient to induce nuclear translocation of the HSF1 isoforms (Fig. 5*B*, *15 min HS panel*). At 2 h after heat shock, we started to detect a cytoplasmic signal for the HSF1γ isoforms, which became more pronounced at 3 h after heat shock (Fig. 5*B*, HSF1γα and HSF1γβ, *2 h rec* and *3 h rec panels*). At both time points, HSF1α and HSF1β were still predominantly localized to the nucleus (Fig. 5*B*, HSF1α and HSF1β, *2 h rec* and *3 h rec panels*). The HSF1γβ isoforms could not be detected in the nucleus from 3 h after heat shock onwards (Fig. 5*B*, HsF1γβ). At 4 h after heat shock, a cytoplasmic signal was also detectable for HSF1α and HSF1β (Fig. 5*B*, *4 h rec panel*). However, HSFα, HSF1β, and HSF1γα still had a rather large fraction of signal visible in the nucleus (Fig. 5*B*, *4 h rec panel*). At 6 h after heat shock, the intracellular distribution of the isoforms was comparable with the non-induced conditions (Fig. 5*B*, compare *basal* and *6 h rec panels*).

##### Hsf1 Isoform Ratio Regulates the Level of Transcription of Heat Shock Protein Genes

**FIGURE 6.**
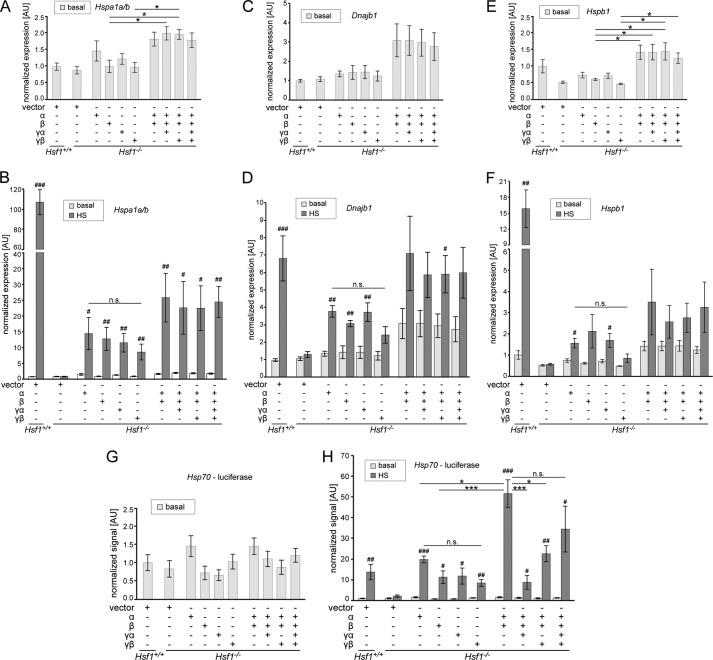
**The transcriptional activities of HSF1 isoforms.** *A–F*, quantitative PCR analysis of HSP transcript levels in wild type (*Hsf1*^+/+^) and *Hsf1* knock-out (*Hsf1*^−/−^) fibroblast lines. Cell lines were transfected with plasmids as indicated below the graphs. Quantitative PCR signals for *Hspa1a/b* (*A* and *B*), *Dnajb1* (*C* and *D*), and *Hspb1* (*E* and *F*) were normalized to the geometric mean of GAPDH and β-actin. Graphs show either the HSP transcript levels of cells that were kept constantly at 37 °C (basal) (*A*, *C*, and *E*) or the basal conditions as before together with the transcript levels 2 h after heat shock (*HS*) (45 min of heat shock, 2 h of recovery at 37 °C) (*B*, *D*, and *F*). The mean expression values are shown normalized to wild type levels (set to 1). Data are mean (*n* > 4) ± S.E. Statistics for basal *versus* heat shock conditions: #, *p* < 0.05, ##, *p* < 0.01, ###, *p* < 0.001. Statistics for comparison of isoforms for each condition: *, *p* < 0.05. *AU* = arbitrary units. *G–H*, *Hsp70* promoter firefly luciferase assay. Cell lines were transfected with plasmids as indicated below the graphs. In addition, thymidine kinase promoter-driven *Renilla* luciferase was added in all conditions to normalize the obtained *Hsp70* promoter firefly luciferase signals. Graphs show either the signals for cells that were kept constantly at 37 °C (*G*, basal) or the basal conditions as before together with the transcript levels 2 h after heat shock (45 min of heat shock, 2 h of recovery at 37 °C) (*H*). The mean signal values are shown normalized to the wild type signal (set to 1). Data are mean (*n* > 3) ± S.E. Statistics for basal *versus* heat shock conditions: #, *p* < 0.05, ##, *p* < 0.01, ###, *p* < 0.001. Statistics for comparison of isoforms for each condition: *, *p* < 0.05, ***, *p* < 0.001. *n.s.* = not significant.

Under basal conditions, expression of *Hspa1a/b* (Hsp70) and *Dnajb1* (Hsp40) was not significantly changed between the *Hsf1* knock out line and wild type cells, and the comparative expression levels did not change on expression of the individual isoforms (Fig. 6, *A* and *C*). Only *Hspb1* (HSP25) showed a significantly lower amount of transcripts in the knock-out line when compared with wild type (Fig. 6*E*; see also Fig. 4*B*). This reduced level could not be rescued by the expression of individual isoforms (Fig. 6*E*). However, expression of combinations of *Hsf1* isoforms under non-induced conditions resulted in higher levels of transcripts for all three HSP genes, when compared with the expression of individual isoforms (Fig. 6, *A*, *C*, and *E*). The amount of DNA used for transfections was kept constant for all conditions, and the relative isoform proportions were (given in %): α and β = 68.7 and 31.3; α, β and γα = 64.2, 29.2, and 6.6; α, β, and γβ = 67.3, 30.6, and 2.1; α, β, γα, and γβ = 62.7, 28.6, 6.6, and 2.1. These proportions were derived from the average levels in mouse peripheral tissues (with testis excluded) as determined in Fig. 2: 6.6% for the *Hsf1*γα isoform and 2.1% for the *Hsf1*γβ isoform. Similar to the induction of heat shock proteins in the wild type fibroblast line (Fig. 4*B*), HSP genes were highly induced 2 h after heat shock (Fig. 6, *Hsf1*^+/+^). Consistent with previous publications (8, 30), *Hsf1* was in all cases required for induction of HSP genes (Fig. 6, compare *Hsf1*^+/+^ vector with *Hsf*^−/−^ vector). However, each individual isoform was sufficient to significantly induce transcription at the *Hspa1a/b* locus after heat shock (Fig. 6*B*). *Hsf1*α, *Hsf1*β, and *Hsf1*γα were sufficient to significantly induce *Dnajb1*(Fig. 6*D*), and for the *Hspb1* gene, *Hsf1*α and *Hsf1*γα were sufficient to significantly induce transcription (Fig. 6*F*). As for non-induced conditions, expression of *Hsf1* isoform combinations resulted in higher levels of transcripts for the respective HSP gene after heat shock when compared with expression of the individual isoforms (Fig. 6, *B*, *D*, and *F*). Although this was not statistically significant, we observed a clear trend for this synergistic enhancement of transcription induction for all three HSP genes (Fig. 6, *B*, *D*, and *F*).

Generally, we noticed a higher variability in transcript levels when *Hsf1* combinations were used, which was more pronounced when the cells were heat-shocked (Fig. 6). This could be the result of slightly different isoform ratios due to different transfection efficiencies between experiments. However, primary fibroblast lines usually show lower transfection efficiencies when compared with commonly used immortalized cell lines, which could also be a contributing factor. To at least partly solve this issue by only analyzing the transfected population of the cells, we used an *Hsp70* promoter luciferase construct to assay the transcriptional activities of the *Hsf1* isoforms. As for the endogenous *Hsp70* locus, we did not observe any significant differences under non-induced conditions for any of the transfection combinations used (Fig. 6*G*). Furthermore, as for the endogenous *Hspa1a/b* gene, expression of the individual *Hsf1* isoforms was sufficient to significantly induce transcription after heat shock (Fig. 6*H*). In this assay, the individual isoforms induced transcription of the luciferase reporter after heat shock to a similar extent as the wild type cell line (Fig. 6*H*, compare *Hsf1*^+/+^ vector with the individual *Hsf1* isoforms). There was no significant difference in luciferase signal between the individual isoforms, although the α isoform showed a trend toward higher activity (Fig. 6*H*, individual isoforms). Expression of a combination of the *Hsf1*α and *Hsf1*β isoform in a ratio of about 2:1 (see above) resulted in a highly significant synergistic induction after heat shock when compared with the signal for the individual isoforms (Fig. 6*H*, compare *Hsf1*α and *Hsf1*β with *Hsf1*α,β co-expression). Interestingly, the addition of either of the *Hsf1*γ isoforms to the *Hsf1*α and *Hsf1*β mixture significantly reduced the signal after heat shock (Fig. 6*H*). The signal for the combination of all four HSF1 isoforms was reduced when compared with the *Hsf1*α and *Hsf1*β combination, although not significantly (Fig. 6*H*). In summary, our data indicate that the ratio of HSF1 isoforms mediates the level of HSP expression in non-induced and heat shock conditions and that HSF1 isoforms can synergistically work to induce transcription of HSP genes.

## DISCUSSION

The mammalian heat shock factor 1 was first cloned 23 years ago (35). It soon became evident that there are two major isoforms created by alternative splicing (21). However, surprisingly little information is available about the molecular functions of HSF1 isoforms, in particular their role in regulation of the heat shock response. We therefore analyzed the molecular functions of HSF1 isoforms and in particular their transcriptional activities during heat shock. Furthermore, we show that two novel HSF1 isoforms exist and are expressed in a wide range of mouse tissues.

We found that the signals for the *Hsf1* isoforms in different tissues were not fully comparable on an absolute level because the GAPDH signals might differ between tissues, However, Fig. 2*C* clearly shows considerable differences in the ratios of the four major isoform transcripts as well as some immature isoforms between tissues. Interestingly, tissues that induce the heat shock response to a much lower extent, such as *e.g.* the brain regions when compared with skeletal muscle (36), have a much lower *Hsf1*α*/Hsf1*β ratio (Fig. 2). They also show more immature *Hsf1* transcripts (Fig. 2*C*). The high levels of immature *Hsf1* transcripts in some tissues (Fig. 2, *e.g.* spleen) open the possibility that HSF1 protein levels are not regulated by changes in transcription rates, but rather through modulation of splicing. This observation is consistent with previous reports that *HSF1* expression is stable and not influenced by stress (25). The ratio of HSF1 isoforms had previously only been studied in heart, brain, and testis (21). Although the ratios for heart and brain are in good agreement with our data, the ratio for testis is different (see Ref. 21 and Fig. 2). In contrast to Goodson and Sarge (21), who observed an *Hsf1*α/*Hsf1*β isoform ratio of about 1 to 2 in testis, we observed an *Hsf1*α/*Hsf1*β isoform ratio of about 4 to 1 in testis (Fig. 2). Unfortunately, it is not easy to compare the data as the sample numbers and error bars have been omitted from the other study (21), although if a difference does exist, it could possibly be due to the different mouse strains used: CBA/J (21) *versus* (CBA/Ca × C57BL/6/J) F1. In this study, we analyzed the *Hsf1* isoform ratios in 8-week-old mice. It would be very interesting to determine whether these ratios change during aging, which could explain some of the changes in the proteostasis capacity of aged tissue (36–42).

Using an antibody against the newly identified exon 9a, which encodes for the 28-amino acid stretch of the two γ isoforms, we showed that the *Hsf1*γ isoforms are indeed translated into protein (Fig. 3). Exon 9a is located between the regulatory domain and the heptad repeat C (Fig. 1*A*). This additional sequence could therefore easily influence the regulation of HSF1 activity, as well as its DNA binding status or protein stability. The activation of HSF1 isoforms after heat shock, which is composed of nuclear import concomitant with post-translational modifications (Figs. 4*C* and 5*B*), seems to be similar between the isoforms. We could detect extensive and comparable post-translational modifications for each isoform as indicated by a hypershift on Western blot (Fig. 4*C*). However, we cannot exclude the possibility that individual isoforms are modified with distinct patterns of post-translational modifications. A decrease in the hypershift begins to occur between 2 and 4 h after heat shock, indicating an attenuation of HSF1 activation (Fig. 4*C*, *2 h* and *4 h rec panels*). At the same time points, the HSF1 isoforms start to appear in the cytoplasm (Fig. 5*B*). Interestingly, for the two HSF1γ isoforms, a cytoplasmic signal is evident by 2 h after heat shock when compared with 4 h for the α and β isoforms (Fig. 5*B*). At 4 h after heat shock, all HSF1 isoforms exhibit a strong cytoplasmic signal, yet they are still to some extent post-translationally modified (compare Figs. 4*C* and 5*B*, *4 h rec panels*). These modifications likely represent inhibitory signals for the transcriptionally active HSF1, *e.g.* phosphorylation on Ser-303 followed by sumoylation on Lys-298 (34). At 6 h after heat shock, all isoforms have returned to the same steady state localization indicative of the non-induced condition (compare Fig. 5*B*, *basal* and *6 h rec panels*). The steady state localization of HSF1 is however somewhat controversial. Very often, predominant nuclear localization of HSF1 has been reported (see for example Ref. 43 and references therein). However, we found that various antibodies against endogenous HSF1 detect a nonspecific staining in *Hsf1* knock-out cells that it predominantly localized to the nucleus, albeit of lower intensity when compared with the wild type cell line (data not shown). Many studies also rely on GFP-fused constructs to detect intracellular localization, with the problem of GFP having an intrinsic propensity to localize to the nucleus (44). Furthermore, many of the cell lines that have been used previously are derived from oncogenic tissue, or are immortalized. These conditions themselves have the potential to induce a relocalization of HSF1 to the nucleus, as for example cancer cells are highly dependent on HSF1 to induce a non-heat shock response transcriptional network (45). Formally, we cannot exclude the possibility that the presence of a FLAG tag and/or the fact that our constructs are overexpressed could also change the localization of the isoforms. However, in the non-induced conditions, only FLAG-tagged HSF1α showed a relatively high fraction of nuclear signal (Fig. 5*B*, α *basal panel*). Taken together, these data are in good agreement with the proposed constitutive nuclear import of HSF1 isoforms (43), balanced by nuclear export and/or degradation (46). The later mechanism has recently been described to quickly attenuate the heat shock response by degrading activated HSF1 (46).

Intriguingly, mouse testes showed a very high level of the *Hsf1*γα isoform, with about 22% of total *Hsf1* levels (Fig. 2*D*). The activation temperature of HSF1 has been shown to be dependent on the average environmental temperature in which the organism lives (47–49), but it is also tissue-specific (47, 50, 51). For example, the threshold temperature to activate HSF1 in male gonad cells is lower than the temperature for the rest of the body (47, 50). Thus, it would be interesting to see whether HSF1γα could be activated at lower temperatures or whether its altered attenuation kinetics (Fig. 5*B*) might contribute to an adaption mechanism to fast changing environmental temperatures.

Knock-out of *Hsf1* does not lead to significantly changed levels of *Hspa1a/b* (HSP70) or *Dnajb1* (HSP40). *Hspb1* (HSP25), however, is down-regulated at both transcript and protein levels (see Ref. 30 and Figs. 4*B* and 6*E*). The expression of many HSP genes depends on the concerted action of multiple transcription factors, *e.g.* members of the V-maf musculoaponeurotic fibrosarcoma oncogene homolog (MAF) family, PAX6, NF-Y, NF-κB, or cAMP-response element-binding protein (CREB) (52, 53). This might explain why the relatively high overexpression of the HSF1 isoforms when compared with the wild type HSF1 amount (see Fig. 4*C*) does not result in elevated levels of HSP transcripts under basal conditions (Fig. 6, basal).

When we analyzed the endogenous levels of *Hspa1a/b* in non-induced cells, we observed a statistically significant higher expression when combinations of isoforms, in particular *Hsf1*α and *Hsf1*β, were expressed (Fig. 6*A*). We also observed a similar pattern of *Dnajb1* expression, although this did not reach statistical significance (Fig. 6*C*). Furthermore, expression of the individual isoforms was not sufficient to rescue the reduced level of *Hspb1* in non-induced conditions (Fig. 6*E*). However, expression of HSF1 isoform combinations restored the basal expression levels of *Hspb1* to be similar to wild type levels (Fig. 6*E*). In general, the HSF1α isoform seems to have the highest transcription activation potential of the four isoforms, although we did not observe statistically significant differences between the individual isoforms (Fig. 6).

We noticed a rather high variability in endogenous HSP gene levels following transfection with the HSF1 isoform combinations, which was most probably at least partly due to unequal ratios in the percentage of transfected fibroblasts. To partly alleviate this problem by analyzing only the transfected subpopulation of the cells, we used an *Hsp70* promoter luciferase assay. Here we did not observe, in contrast to the analysis of endogenous *Hspa1a/b* (Fig. 6*A*), a synergistic effect for HSF1 isoform combinations under basal conditions (Fig. 6*G*). However, 2 h after heat shock, we saw a dramatic, highly statistically significant synergistic enhancement when expressing HSF1α and HSF1β together when compared with the expression of only the individual isoforms (Fig. 6*H*). It is intriguing that the average ratio of *Hsf1*α to *Hsf1*β is ∼2 to 1 (Figs. 2 and 4*A*). Therefore, stochastically each HSF1β isoform could heterotrimerize with two HSF1α isoforms. This 2 to 1 ratio could potentially create a different structure in the trimeric transcription activation domain and thereby influence HSF activation, for example by inhibiting binding of HSP70 and HSP40 (54) or by forming a platform for interaction with other transcriptional regulators (55, 56).

During stress and development, HSF1 can form heterodimers with HSF2 and possibly other HSFs (57). Furthermore, a recent study that analyzed HSF1 and HSF2 isoforms, mainly under normal conditions or proteasome inhibition, suggested that the HSF2β/HSF2α ratio influences HSF1 activity, in particular HSF1β (8). Thus, HSF heterotrimer formation or changes in the expression pattern of HSF2 isoforms are most probably additional determinants of the level of the heat shock response.

In our luciferase assay, the addition of the HSF1γ isoforms seemed to have an inhibitory effect on transcriptional activation (Fig. 6*H*) by almost completely abolishing the synergistic effects of *Hsf1*α and *Hsf1*β co-expression. Such antagonistic functions of isoforms generated from the same transcript have been previously described for other transcription factors, *e.g.* acute myeloid leukemia 1 (AML1) (58) and cAMP-responsive-element modulator (CREM) (59). Hence, if this inhibitory effect of the γ isoforms can be confirmed in further experiments (other cell lines, different HSP genes, etc.), the varying expression of HSF1γ isoforms in different tissues (Fig. 2) could be a major determinant of the HSP mosaic of each tissue type.

Taken together, our data provide proof for the existence of two novel HSF1 isoforms, which are expressed across a wide range of mouse tissues. We have also shown for the first time that the ratio of HSF1 isoforms determines the rate of heat shock protein gene transcription, under both non-stressed and heat shock conditions. Furthermore, expression of isoforms in a distinct ratio leads to a synergistic enhancement of transcription of HSP genes. In contrast, the physiological function of the HSF1γ isoforms might be to attenuate the level of transcriptional activation by the two major isoforms, HSF1α and HSF1β. We propose that HSF1 isoform ratios are a major factor in the regulation of the expression of heat shock protein genes and that modulation of this ratio could influence the stress response capacity of various tissues.

## Footnotes

3 The abbreviations used are:

HSF1: heat shock transcription factor 1
α: Hsf1α isoform
β: Hsf1β isoform
γα: Hsf1γα isoform
γβ: Hsf1γβ isoform
HSP: heat shock protein
rec: recovery
IP: immunoprecipitation
FAM: 6-carboxyfluorescein
TAMRA: 6-carboxytetramethylrhodamine.
